# Non-Invasive Respiratory Support in “De Novo” Acute Hypoxemic Respiratory Failure: Which Technique Is Best?

**DOI:** 10.3390/medicina62050805

**Published:** 2026-04-22

**Authors:** Paolo Groff, Stefano De Vuono

**Affiliations:** Emergency Department, Santa Maria della Misericordia Hospital, 06129 Perugia, Italy; stefano.devuono@ospedale.perugia.it

**Keywords:** “de novo” respiratory failure, non-invasive respiratory support, patient self-inflicted lung injury

## Abstract

*Background*: One of the most debated scientific topics in recent years is the role of non-invasive respiratory support techniques in the treatment of de novo acute hypoxemic respiratory failure. Until pre-COVID-19, the most accredited guidelines did not make recommendations for or against the use of these techniques in this clinical condition, and the increased risk of adverse events for patients who failed the non-invasive approach was widely reported in the literature. The most recent guidelines recommend the use of HFNC as a first-line technique in the treatment of de novo acute hypoxemic respiratory failure to avoid the need for tracheal intubation. However, the strength of these recommendations remains weak, the quality of the underlying evidence is poor, and their usefulness in deciding which technique to apply to an individual patient is questionable. *Aim*: The aim of this review was to provide the reader with some critical tools to interpret the different indications regarding the choice of the best non-invasive support technique to be used in this setting. *Methods*: To this end, we analyzed the available literature on this topic, privileging the works that are most useful in correlating the practical indications to the pathophysiological assumptions. *Results and Conclusions*: The notable heterogeneity of the studies on which the current recommendations are based, as well as the affirmation of the concept of patient self-induced lung injury (P-SILI), highlights the importance of assessing each patient’s risk of developing this complication, individualizing treatment to the patient’s specific needs, and monitoring the patient during treatment.

## 1. Introduction

We would like to begin this review with a clinical case. Imagine a young man presents to your emergency department with fever and worsening dyspnea. His medical history is unremarkable. On examination, the patient is alert and cooperative, but exhibits reduced breath sounds and crackles in the right lower lung field, with mottled skin.

Vital signs: an arterial pressure of 100/55; a respiratory rate of 38/min; a heart rate of 120/min; a GCS of 15/15; and a body temperature of 39 °C

Blood gas analysis: pH 7.28; a PaO_2_ level of 45; a PaCO_2_ level of 38; an HCO_3_^−^ level of 17; and a lactate level of 4.1

This patient is suffering from “de novo” acute hypoxemic respiratory failure due to severe pneumonia.

Regarding the definition, the joint guidelines of the French-speaking Society of Resuscitators and the French Emergency Medicine Society on oxygen therapy in acute respiratory failure define acute hypoxemic respiratory failure type 1 as a condition in which the lung is unable to oxygenate mixed venous blood, occurring “de novo”. This excludes hypoxemia associated with acute cardiogenic pulmonary edema or exacerbations of chronic lung disease [1]. In the majority of cases, the underlying cause is pneumonia. Although this definition has been used for years, many studies concerning the use of non-invasive ventilation techniques in hypoxemic respiratory failure have yielded inconsistent results. These studies often included significant percentages of patients with hypoxemia secondary to heart failure or chronic obstructive pulmonary disease exacerbations, who respond differently to these techniques. This has variably led to an overestimation of the efficacy of these methods in some studies [2]. Hence, it is important to provide a standardized definition to better characterize patients in whom hypoxemia arises acutely (“de novo”) rather than as an aggravation of a preexisting cardio-pulmonary condition. We define type 1 hypoxemic respiratory failure as PaO_2_ < 60 mmHg and/or SpO_2_ < 90% breathing room air or a PaO_2_/FiO_2_ ratio lower than 300 mmHg. Clinical signs include a respiratory rate above 25/minute, accessory respiratory muscle activation, abdominal paradox, cyanosis, and dyspnea [2]. The treatment of hypoxemia, per se, consists of oxygen administration; hypoxemia associated with clinical signs of respiratory distress requires non-invasive respiratory support.

Which respiratory support technique would you choose for this patient? Endotracheal intubation, conventional oxygen therapy (COT), high-flow nasal cannulas (HFNC), or NIV? Until a few years ago, the ATS/ERS 2017 guidelines did not provide recommendations for certain clinical conditions, including “de novo” acute respiratory failure, acute asthma, or pandemic viral infections [3]. Many factors have since changed our approach to “de novo” acute respiratory failure and fueled the debate surrounding non-invasive respiratory support. These include the adoption of a unified definition of “de novo” respiratory failure; the widespread use of HFNC; the COVID-19 pandemic; the publication of new randomized controlled trials (RCTs); the drafting of subsequent guidelines; the consolidation of the pathophysiological concept of patient self-inflicted lung injury (P-SILI); and the growing consensus that treatment must be individualized.

The aim of this review is to discuss the use of respiratory support techniques for “de novo” acute hypoxemic respiratory failure, bridging pathophysiological assumptions with clinical practice. By the end of this review, it will be clear that, although there are no unequivocal indications in the currently available literature, given the great heterogeneity of studies, the best approach in this type of patient is to tailor the type of support to the patient’s pathophysiological characteristics.

## 2. Methods

A comprehensive electronic search was performed via the PubMed database to identify papers published through March 2026 regarding the effectiveness of COT, HFNC, and NIV in the management of acute hypoxemic respiratory failure. The inclusion criteria were full-text availability, English language, human subjects, peer-reviewed status, and classification as RCTs, observational studies, or systematic reviews. The articles primarily focused on hypercapnic respiratory failure (e.g., COPD exacerbations and acute heart failure), editorials, case reports, preclinical studies, and non-peer-reviewed pre-prints were excluded. The following Boolean phrase was used: (“High-Flow Nasal Cannula” OR “HFNC”) AND (“Noninvasive Ventilation” OR “NIV”) AND (“Acute Hypoxemic Respiratory Failure” OR “pneumonia”) AND (“Patient’s Self Inflicted Lung Injury” OR “P-SILI”). Discrepancies regarding article inclusion were resolved through discussion between reviewers.

## 3. The Increasing Use of High-Flow Nasal Cannulas (HFNCs)

As mentioned, HFNC has emerged as a major non-invasive respiratory support technique. It delivers high flows of preheated and humidified air–oxygen mixtures through a nasal interface. Physiological studies conducted approximately a decade ago demonstrated that, due to high flow rates, this method provides a FiO_2_ not influenced by the patient’s breathing pattern. Furthermore, humidification and preheating reduce airway resistance. Finally, the high flow rates provide a modest PEEP effect and, crucially, ensure excellent dead-space washout. Consequently, various studies have demonstrated that HFNC, while offering oxygenation capacity comparable to positive pressure, significantly reduces inspiratory effort and improves patient comfort [4,5,6,7,8].

The 2015 study by Frat et al. significantly influenced clinical practice, demonstrating substantially equal efficacy for HFNC, COT, and NIV in preventing endotracheal intubation and 28-day mortality [9]. Interestingly, 90-day mortality was better with HFNC, but more importantly, a sub-analysis of the most severely ill patients, i.e., those with a PaO_2_/FiO_2_ ratio of less than 200 mmHg, demonstrated that in this patient category, HFNC was more effective than the other two methods in preventing intubation within 28 days. Interestingly, the patients who demonstrated a worse outcome were those who, despite respiratory support, maintained a high tidal volume, demonstrating persistent high inspiratory effort, as we will discuss later.

Regarding the use of non-invasive respiratory support methods, there is no doubt that the COVID-19 pandemic has had a significant impact. During the pandemic, there was an increase in the use of all these techniques, and HFNC in particular [10]. However, their use was often driven by the need to match available resources to the enormous growth in demand and the resulting shortage of intensive care beds, rather than by a rational indication. During this phase, numerous studies were produced that were of poor quality and, in any case, difficult to compare in terms of the methods used, organizational settings, and patient populations studied [11].

## 4. Non-Invasive Respiratory Support: Clinical Trials and Guidelines

A fair number of randomized, controlled clinical trials have been conducted in parallel or following Frat’s work (Table 1). Although there is some heterogeneity among the various studies, especially in terms of methods and populations studied, as some of them were dedicated to COVID-19 patients, and others to immunosuppressed patients, we can summarize their results as follows. HFNC has proven more effective than COT in preventing endotracheal intubation, while no benefit has been observed with respect to in-hospital mortality. These data do not change with COVID-19 status or immunosuppression and are confirmed by a recently published multicenter prospective trial by Frat and co-workers [9,12,13,14,15,16,17,18]. There are no significant differences in terms of intubation or mortality between CPAP/NIV and COT [9,19,20,21]; in one study, CPAP proved more effective in preventing intubation in COVID-19 patients [12]. No significant differences in terms of intubation or mortality were observed between HFNC and NIV [9,22,23]; in one study, helmet NIV with higher PEEP was more effective than HFNC in preventing intubation in COVID-19 patients [24]. Even the recent RENOVATE study, which enrolled approximately 1800 patients at 33 hospitals in Brazil and aimed to demonstrate the non-inferiority of HFNC to NIV with respect to the primary outcome of intubation/seven-day mortality and a series of other secondary and tertiary outcomes, including patient comfort, showed no significant differences between the two methods in non-immunosuppressed patients with AHRF and in hypoxemic COVID-19 patients in the primary analysis and sensitivity analysis. Interestingly, among the tertiary outcomes, HFNC was superior to NIV with respect to comfort [25] (Table 1).

Based on this evidence, a series of guidelines have been produced by various scientific societies. Due to their importance, we cite here the document produced by the European Society of Intensive and Critical Care Medicine (ESICM) and the joint guidelines of the French-speaking Society of Intensive Care Physicians and the French Society of Emergency Physicians [3,26]. As can be expected, there are differences in the recommendations, but in summary, both recommend the use of HFNC rather than COT to prevent intubation, without providing any indication regarding mortality. There are no recommendations regarding the use of CPAP/NIV rather than COT with respect to either intubation or mortality; the ESICM guidelines provide a weak recommendation in favor of CPAP to prevent intubation in COVID-19 patients. The ESICM does not provide recommendations on the use of NIV rather than HFNC with respect to either intubation or mortality, but does provide a weak recommendation in favor of NIV to prevent intubation in COVID-19 patients. The French guidelines, however, consider that HFNC should be used instead of NIV in the AHRF (Table 2).

In conclusion, the recommendations by current guidelines are based on heterogeneous studies and provide contradictory indications, particularly when physicians have to choose which NIRS modality applies to an individual patient. Due to different methodologies, the heterogeneity of the populations included, and the different outcomes considered, even in the most recent clinical studies, randomized controlled trials (RCTs) have produced different results, with some studies showing that HFNC may be preferred over NIV to treat AHRF, and others suggesting that different interfaces and settings may affect results. However, one aspect on which there is unanimous agreement is the fact that delaying a needed intubation increases mortality [11].

## 5. Bridging Pathophysiology and Clinical Practice

And this brings us to the topic of patient self-inflicted lung injury (P-SILI), a pathophysiological concept that has been developing over the past few years. Preserving spontaneous breathing offers advantages, such as avoiding the side effects of mechanical ventilation and deep sedation and maintaining aeration in dependent lung areas [27]. However, maintaining spontaneous breathing can be associated with dysregulated inspiratory effort, resulting in tachypnea and excessive tidal volumes. This leads to increased pulmonary stress and strain, as well as to the uneven distribution of the force produced by the diaphragm within the lung due to the uneven distribution of the damage, resulting in the so-called pendelluft effect. Furthermore, increased transmural pressure in the pulmonary capillaries can lead to pulmonary edema, and ultimately, the contractile action of the diaphragm becomes uneven, having to act on lung regions with different compliance, leading to myotrauma and diaphragmatic weakness [27]. This mechanism has been described as a vicious cycle process in which lung damage evokes an increase in respiratory drive, which, in turn, generates further lung damage, and so on [28,29,30,31,32]. It is also clear that positive pressures, synchronizing with the increased respiratory drive, can contribute to an increase in tidal volumes and, consequently, to pulmonary stress and strain [33]. Finally, it is clear that several studies indicate a relationship between the severity of the underlying lung disease and the intensity of inspiratory effort and vice versa, explaining the extreme variability of this phenomenon in different patients [34]. It is then possible that the presence or absence of this dysregulation of inspiratory effort determines the response to non-invasive respiratory support techniques and the possibility of an unfavorable outcome in predisposed patients [35,36].

One of the major determinants of pulmonary stress and strain, which causes increased tidal volumes, is transpulmonary pressure, which is the pressure that maintains lung distension and is the algebraic sum of intrapleural and intra-alveolar pressures. Now, applying pressure support increases intra-alveolar pressure, but if this method is unable to reduce intrapleural pressure in the presence of increased respiratory distress, the transpulmonary pressure can only increase further, exposing the patient to an increased risk of P-SILI. HFNC has been shown to negligibly increase intra-alveolar pressure, while in some patients, it may reduce the intensity of inspiratory effort and, therefore, intrapleural pressure [27]. This may explain why HFNC may be more effective than NIV in some patients.

While numerous experimental studies demonstrate the validity of the P-SILI concept, many clinical studies provide indirect validation [37]. As already mentioned, in Frat’s study, patients who maintained high tidal volumes had the worst outcomes in terms of intubation and mortality, while other studies demonstrated that persistent high inspiratory effort despite non-invasive support increased the risk of failure [9]. Finally, as already mentioned, Yoshida and coworkers demonstrated a one-to-one relationship between clinical severity and intensity of inspiratory effort and, consequently, exposure to the risk of P-SILI [34]. Therefore, the intensity of inspiratory drive and effort becomes an important determinant of the success of non-invasive support methods in each individual patient, hence the need to monitor it and, if possible, modulate it with an approach we might define as a tailoring intervention to the patient’s needs [12,37,38,39].

## 6. Discussion: How to Choose the Right Method for the Right Patient?

The decision on which technique to apply and the duration of the attempt must, therefore, be made considering the severity of the underlying condition, including the compromised oxygen exchange and the patient’s neurological and hemodynamic status. This must be followed by monitoring gas exchange, as well as inspiratory effort, using tidal volumes, where available, esophageal manometry, or its surrogates such as nasal pressure, central venous pressure swings, or clinical indices of respiratory distress, or diaphragmatic ultrasound, composite scores, or diaphragmatic impedance analysis, depending on the patient’s intensity and organizational setting.

**Figure 1:**
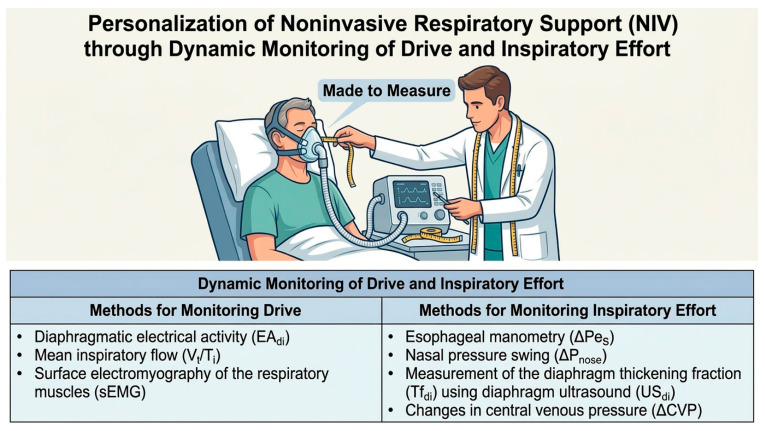
Dynamic monitoring of drive and respiratory effort.

Given the need to monitor a patient’s inspiratory drive and effort, it is important to understand the advantages and limitations of each method, as this also affects the type of patient treated in different organizational settings. Clinical signs of distress are easily accessible and can even be assessed semi-quantitatively, but they are difficult to standardize and can yield misleading results. During non-invasive respiratory support, the best way for monitoring respiratory drive is measurement of diaphragmatic electrical activity (EAdi) [40], a reliable measure of respiratory drive, where individual changes over time provide more information than static measurements; however, this technique is invasive, expensive, and without validated reference values. Alternative methods for assessing respiratory drive during spontaneous breathing are the mean inspiratory flow (Vt/Ti) [41], which reflects the speed of lung expansion and, therefore, the balance between neuromuscular drive and the mechanical properties of the respiratory system, but which could underestimate in subjects with muscular weakness [39], and respiratory muscle surface electromyography (sEMG), which consists of the transcutaneous measurement of the activity of the respiratory muscles, but it is mainly limited by the difficulty in ensuring that the recorded signal comes specifically from the diaphragm [42]. The gold standard for monitoring inspiratory effort is represented by esophageal manometry and, in particular, by its derivative Pmusc, which also takes into account the elastic and resistive load of the chest wall and whose variations between respiratory acts could reliably reflect the variations in inspiratory effort, but it is an expensive and invasive technique [39,43]. Nasal pressure swing (ΔPnose) is one of the most promising surrogate techniques for monitoring inspiratory effort which, through a pressure transducer inserted in the nostril, reflects the variations in alveolar pressure and correlates well with variations in transesophageal pressure; the main advantage is that it can be easily measured in spontaneously breathing patients, both during NIV and HFNC, but its main limitation is that the patient must breathe with their mouth closed [44]. The measurement of the diaphragm thickening fraction (Tfdi) using diaphragm ultrasound (USdi) is certainly the surrogate method for monitoring diaphragm effort over time that is most easily performed at the bedside in spontaneously breathing patients; however, its main limitations are that it provides a one-dimensional study of the diaphragm and that its measurement could be distorted by passive displacement of the diaphragm during the application of positive pressures [39,45]. In patients who have had a central venous catheter placed, measuring changes in central venous pressure can be a good alternative method for monitoring diaphragmatic effort, provided that the patient’s intravascular volume and cardiac function are taken into account [46] (Figure 1 and Table 3).

In addition to these innovative techniques, it is important to remember that more ‘traditional’ parameters can also provide valuable information for monitoring the drive and inspiratory effort. The first of these is represented by tidal volume. A high tidal volume generally corresponds to a high inspiratory effort, so a reduction in tidal volume can be a sign of decreased inspiratory effort. Conversely, an increase in tidal volume may have been caused by excessive support pressures [39]. Additionally, variations in tidal volume in response to the application of positive pressures allow us to distinguish between patients with an active breathing pattern, who, therefore, benefit from increasing support pressures, and patients with a passive pattern, in whom the application of excessive support pressures can generate excessive increases in transpulmonary pressure [47]. Also, changes in the severity of dyspnea and the extent of respiratory rate are useful parameters to include in the monitoring of patients with acute de novo respiratory failure [39]. Finally, in addition to the trend over time of the well-known PaO_2_/FiO_2_ ratio, the presence of hypocapnia and its trend in response to the application of respiratory support can also be useful to understand whether a patient’s respiratory effort is decreasing. It is well known that hypocapnia reflects a state of hyperventilation, both in terms of respiratory rate and depth, and it has been shown that patients with de novo acute respiratory failure who are hypocapnic benefit from the application of support pressures compared with non-hypocapnic patients [24] and that hypocapnia can predict the severity of de novo respiratory failure in certain categories of patients, such as COVID-19 patients [48].

Following these concepts and, above all, the need to individualize patient intervention, some authors have proposed the concept of “lung-protective non-invasive respiratory support,” which involves a multidimensional approach. It involves monitoring patient discomfort with appropriate tools, the extent of inspiratory effort with the tools we just discussed, and the application of nonpharmacological or pharmacological strategies aimed at achieving respiratory drive control and conscious sedation [38].

This phase is followed by the selection of the most appropriate respiratory support device for the individual patient. A recent systematic review of all studies analyzing the physiological effects of different non-invasive respiratory support techniques (COT, HFNC, CPAP, and NIV) found that HFNC has the least impact on transpulmonary pressure compared with other techniques and is, therefore, less likely to cause lung injury from spontaneous breathing [49]. Meanwhile, in the presence of dysregulated and excessive respiratory drive, this technique may be insufficient to relieve the patient from respiratory distress and the consequent onset of P-SILI. Conversely, NIV is the technique that most reduces the work of breathing, despite having a significant impact on transpulmonary pressure. It may, therefore, reduce inspiratory effort where this is excessive, but it may also increase the severity of lung injury if not adequately controlled.

Some authors propose the following systematic approach to a patient with “de novo” hypoxemic acute respiratory failure potentially eligible for treatment with non-invasive respiratory support [50,51]: (1) Evaluate the patient’s severity by considering the value of the PaO_2_/FiO_2_ ratio; the overall disease severity, possibly using scores such as the SOFA or MEWS; the state of consciousness; and the need for vasopressors. (2) On the basis of this information, evaluate the risk of failure of the method. Numerous data in the literature correlate the risk of failure with the severity of the PaO_2_/FiO_2_ ratio and the patient’s clinical conditions, including comorbidities [24,52,53]. If the PaO_2_/FiO_2_ ratio is less than 100 mm Hg and the patient’s severity indicates a high probability of failure, do not start any non-invasive treatment, as the patient will have to be intubated and mechanically ventilated. If, despite the severity of hypoxemia, the clinical picture suggests a low probability of failure, it is advisable to proceed with a brief attempt with a non-invasive method, lasting a maximum of 1 or 2 h. It is advisable to initiate non-invasive treatment in a patient with a PaO_2_/FiO_2_ ratio of at least 150 mmHg, adjusting the duration of the trial according to the severity of the organ compromise (1–2 h if high; 3–6 h in milder conditions). (3) The subsequent choice of method, again, depends on the patient’s condition. In the presence of a PaO_2_/FiO_2_ of less than 200 and the patient’s inspiratory effort being significant, the preferred technique is helmet NIV; in patients with a PaO_2_/FiO_2_ greater than 200, in whom respiratory distress appears moderate according to detection techniques, it is possible to begin with HFNC [49]. (4) The final step is to closely monitor the patient by implementing all the methods at our disposal and with which we are most familiar. Is the time trend of the monitoring parameters that we have chosen and the integration of the information that every single parameter can give us the best way to understand if we are effectively supporting our patient’s respiratory effort, or if our respiratory support is failing? All this must be considered while always keeping in mind not to delay intubation in patients in whom we are failing and who need it, because one of the fundamental points against the use of positive pressures in spontaneous breathing is the fact that delaying intubation in these patients can mean negatively marking the prognosis [54]. It must be reiterated that this patient-tailored approach is neither standardized nor based on clear scientific evidence, but it is what most experts on the subject currently recommend.

## 7. Conclusions

In conclusion, current guidelines do not provide clear indications on what is the best non-invasive respiratory support in patients with acute de novo respiratory failure. While recommending the preferential use of HFNC, the heterogeneity among studies and the overall quality do not allow us to provide certain indications on how to support more serious patients. However, in daily clinical practice, physicians often try to support these patients with positive pressure in a non-invasive way in order to avoid intubation. This lack of clear indications derives, in fact, from the lack of solid data in the literature. At the same time, there is increasing awareness of the existence of P-SILI and how we can understand which of these patients are at risk or are developing such self-induced lung damage. To this end, an ever-increasing number of useful methods have been proposed, and attempts are being made to monitor both drive and respiratory effort in spontaneously breathing patients. In the current state, the most correct approach in patients with acute de novo respiratory failure would seem to be to first evaluate the initial severity of the patient and to understand what the objective and the best program are for the patient we are treating, along with strict continuous monitoring.

## Figures and Tables

**Table 1:** Main clinical trials comparing the efficacy of different non-invasive respiratory techniques as to the outcomes of need for intubation and in-hospital mortality in acute hypoxemic “de novo” respiratory failure. HFNC: high-flow nasal cannulae; COT: conventional oxygen therapy; CPAP: continuous positive airway pressure; NIV: non-invasive ventilation.

Comparison	Main Trials	Outcomes
**HFNC vs. COT**	• Frat 2015 [9] • Frat 2026 [18] • Perkins 2022 [12] (COVID-19) • Bouadma 2022 [17] (COVID-19) • Azoulay 2018 [13] (immunocompromised) • Ospina-Tascón 2021 [14] (COVID-19) • Frat 2022 [15] (COVID-19) • Alpekinoglu Mendil 2021 [16] (immunocompromised)	• 30-day mortality: no statistical difference • 28-day intubation: beneficial effect favoring HFNC • No difference in treatment effect based on COVID-19 or immunocompromised status
**CPAP/NIV vs. COT**	• Frat 2015 [9] • Perkins 2022 [12] (COVID-19) • Brambilla 2014 [19] • Lemiale 2015 [20] (immunocompromised) • He 2019 [21]	• No significant effect of CPAP/NIV on intubation or in-hospital mortality • Lower intubation with CPAP in COVID-19 (one study)
HFNC vs. NIV *	• Frat 2015 [9] • Nair 2021 [22] (COVID-19) • Grieco 2021 [24] (COVID-19) • Coudroy 2022 [23] (immunocompromised)	• No significant difference in in-hospital mortality or intubation • One study favoring helmet NIV to prevent intubation in COVID-19 patients

* No study has compared CPAP with HFNC.

**Table 2:** Synthesis of the recommendations of the ESICM and the SRLF-SFMU guidelines. HFNC: high-flow nasal cannulae; COT: conventional oxygen therapy; CPAP: continuous positive airway pressure; NIV: non-invasive ventilation.

	HFNC vs. COT	CPAP/NIV vs. COT	HFNC vs. COT
**ESICM guidelines on acute respiratory distress syndrome: definition, phenotyping and respiratory support strategies (2023) ** **(Reference** [26] **)**	• HFNC recommended over COT to prevent intubation (strong recommendation; moderate level of evidence in favor) • No recommendation (in favor or against) to prevent in-hospital mortality	• No recommendation (in favor or against) to prevent intubation or in-hospital mortality • Weak recommendation in favor of CPAP to prevent intubation in COVID-19 pts	• No recommendation in favor or against to prevent intubation or in-hospital mortality • Weak recommendation in favor of CPAP/NIV to prevent intubation in COVID-19 patients
**Oxygen therapy in acute hypoxemic respiratory failure: guidelines from the SRLF-SFMU consensus conference (2024) ** **(Reference** [3] **)**	• HFNC should probably be used rather than COT in HARF pts with an O_2_ flow rate > 6 L/m to achieve a SpO_2_ > 92% or a PaO_2_/FiO_2_ < 200 (GRADE 2+, moderate quality of evidence, and strong agreement)	• No recommendation concerning the use of CPAP rather than COT in acute hypoxemic respiratory failure patients • No recommendation concerning the use of NIV vs. COT in de novo acute hypoxemic respiratory failure including immunocompromised patients	• HFNC should probably be used rather than NIV in pts with de novo acute hypoxemic respiratory failure (GRADE 2+, moderate quality of evidence, and strong agreement)

**Table 3:** Techniques for monitoring respiratory drive and respiratory effort.

		Advantages	Limitations
**Techniques f** **or monitoring respiratory drive**	**Diaphragmatic electrical activity (EAdi)**	Reference method	Invasive, expensive and without validated reference values
	**Mean inspiratory flow (Vt/Ti)**	Non-invasive; easily derived from ventilator waveforms in non-assisted patients	Could underestimate in subjects with muscular weakness
	**Respiratory muscle surface electromyography (sEMG)**	Non-invasive	Difficulty in ensuring that the recorded signal comes specifically from the diaphragm
**Techniques for monitoring inspiratory effort**	**Esophageal manometry (ΔPES and Pmusc)**	Reference method	Invasive and expensive
	**Nasal pressure swing (ΔPnose)**	Easily measured in spontaneously breathing patients	Patient must breathe with their mouth closed
	**Diaphragm thickening fraction (Tfdi) using diaphragm ultrasound (USdi)**	The most easily performed at the bedside in spontaneously breathing patients	One-dimensional study of the diaphragm; its measurement could be distorted by passive displacement of the diaphragm
	**Central venous pressure swing ( ΔCVP)**	Easily measured	Influenced by intravascular volume and cardiac function

## Data Availability

This work did not produce any new data.
